# Adding an Artificial Tail—Anchor to a Peptide-Based HIV-1 Fusion Inhibitor for Improvement of Its Potency and Resistance Profile

**DOI:** 10.3390/molecules22111996

**Published:** 2017-11-20

**Authors:** Shan Su, Zhenxuan Ma, Chen Hua, Weihua Li, Lu Lu, Shibo Jiang

**Affiliations:** 1 Key Laboratory of Medical Molecular Virology of MOE/MOH, School of Basic Medical Sciences & Shanghai Public Health Clinical Center, Fudan University, 130 Dong An Rd., Xuhui District, Shanghai 200032, China; sushan1666@gmail.com (S.S.); zxma13@fudan.edu.cn (Z.M.); 16211010047@fudan.edu.cn (C.H.); 2 Key Laboratory of Reproduction Regulation of National Population and Family Planning Commission, The Shanghai Institute of Planned Parenthood Research, Institute of Reproduction and Development, Fudan University, Shanghai 200032, China; iamliweihua@foxmail.com; 3 Lindsley F. Kimball Research Institute, New York Blood Center, New York, NY 10065, USA

**Keywords:** HIV, gp41, fusion inhibitor, six-helix bundle, peptide

## Abstract

Peptides derived from the C-terminal heptad repeat (CHR) of human immunodeficiency virus type 1 (HIV-1) envelope protein transmembrane subunit gp41, such as T20 (enfuvirtide), can bind to the N-terminal heptad repeat (NHR) of gp41 and block six-helix bundle (6-HB) formation, thus inhibiting HIV-1 fusion with the target cell. However, clinical application of T20 is limited because of its low potency and genetic barrier to resistance. HP23, the shortest CHR peptide, exhibits better anti-HIV-1 activity than T20, but the HIV-1 strains with E49K mutations in gp41 will become resistant to it. Here, we modified HP23 by extending its C-terminal sequence using six amino acid residues (E6) and adding IDL (Ile-Asp-Leu) to the C-terminus of E6, which is expected to bind to the shallow pocket in the gp41 NHR N-terminal region. The newly designed peptide, designated HP23-E6-IDL, was about 2- to 16-fold more potent than HP23 against a broad spectrum of HIV-1 strains and more than 12-fold more effective against HIV-1 mutants resistant to HP23. These findings suggest that addition of an anchor–tail to the C-terminus of a CHR peptide will allow binding with the pocket in the gp41 NHR that may increase the peptide’s antiviral efficacy and its genetic barrier to resistance.

## 1. Introduction

Human immunodeficiency virus (HIV) is the causative pathogen of acquired immune deficiency syndrome (AIDS). So far, 35 individual anti-HIV drugs and five combination formulas have been approved for clinical use by the U.S. Food and Drug Administration (FDA). According to the Joint United Nations Programme in HIV and AIDS (UNAIDS), only 82% of HIV/AIDS patients on treatment had suppressed viral loads at the time of the survey (http://www.unaids.org/en/resources/documents/2017/20170720_Global_AIDS_update_2017), meaning that about 3.5 million HIV-infected patients receiving antiviral treatment showed no control of their disease progress. One major reason is that HIV-1 rapidly mutates during treatment and quickly acquires resistance to the anti-HIV drugs used. Most anti-HIV drugs have been reported to induce drug-resistant HIV-1 strains within several weeks to several years after drug treatment [1,2,3]. More surprisingly, in a 2016 Mexican survey, about 14.4% of HIV-1 strains isolated from HIV patients not treated with anti-HIV drugs showed pretreatment resistance to any antiretroviral drug [4]. Meanwhile, pretreatment resistance to antiretroviral drugs has been reported in many countries [5,6,7]. Therefore, development of anti-HIV drugs with high genetic barrier to resistance and high sensitivity to currently circulating drug-resistant HIV-1 strains is urgently needed.

Among the 35 anti-HIV drugs, only two HIV-1 entry inhibitor-based anti-HIV drugs, enfuvirtide (T20 peptide) and maraviroc (CCR5 antagonist), can block HIV-1 fusion with and entry into the target cell. During the process of HIV type 1 (HIV-1) entry into the target cells, the viral envelope glycoprotein (Env) surface subunit gp120 binds to receptor CD4 and co-receptor (CCR5 or CXCR4) on the target cell. After that, the C-terminal heptad repeat (CHR) of the HIV-1 Env transmembrane subunit gp41 interacts with the gp41 N-terminal heptad repeat (NHR) to form the six-helix bundle (6-HB), in which three NHRs form a trimer core and three CHRs bind to the hydrophobic grooves on the trimer surface in an antiparallel way [8,9]. The 6-HB pulls the membranes of HIV-1 and target cell together for fusion. Maraviroc can block HIV-1 binding to the cellular co-receptor CCR5, while T20 can bind to the gp41 NHR trimer to block the formation of 6-HB. However, the clinical application of these two entry inhibitors is limited because both can induce drug-resistant mutants in the treated HIV/AIDS patients [10,11]. For example, HIV-1 strains with mutations at the inhibitor-binding sites in the gp41 NHR region, such as the GIV motif and the hydrophobic pocket formed by Gly547-Leu556, became resistant to T20 and other CHR peptides [12,13,14,15,16,17,18]. The next-generation peptidic fusion inhibitors with significantly improved anti-HIV-1 activities, including sifuvirtide (SFT) [19], TRI-1144 (T2635) [20], and HP23, the shortest (23 residues) CHR peptide with potent HIV fusion inhibitory activity [21], also induced drug resistance in vitro [22,23,24,25,26]. Therefore, developing novel HIV fusion inhibitors with higher genetic barriers to resistance still remains urgent.

We have previously demonstrated that addition of an IDL (Ile-Asp-Leu) anchor to the C-terminus of a CHR peptide could improve the peptide’s anti-HIV-1 activity [27]. Here, we modified HP23 by extending its C-terminal sequence using six residues (E6) and adding IDL to the C-terminus of E6, which is expected to bind to the shallow pocket in the N-terminal region of the gp41 NHR-trimer. The newly designed peptide, designated HP23-E6-IDL, was about 2.0- to 15.8-fold more potent than HP23 against a broad spectrum of HIV-1 strains and 1.9- to 20.7-fold more effective against HIV-1 mutants resistant to T20, T2635, and HP23, suggesting that this approach can be applied to the design of peptide-based viral fusion inhibitors with improved antiviral efficacy and resistance profiles.

## 2. Results

### 2.1. HP23-E6-IDL Formed Stable 6-HB With N46

**Figure 1:**
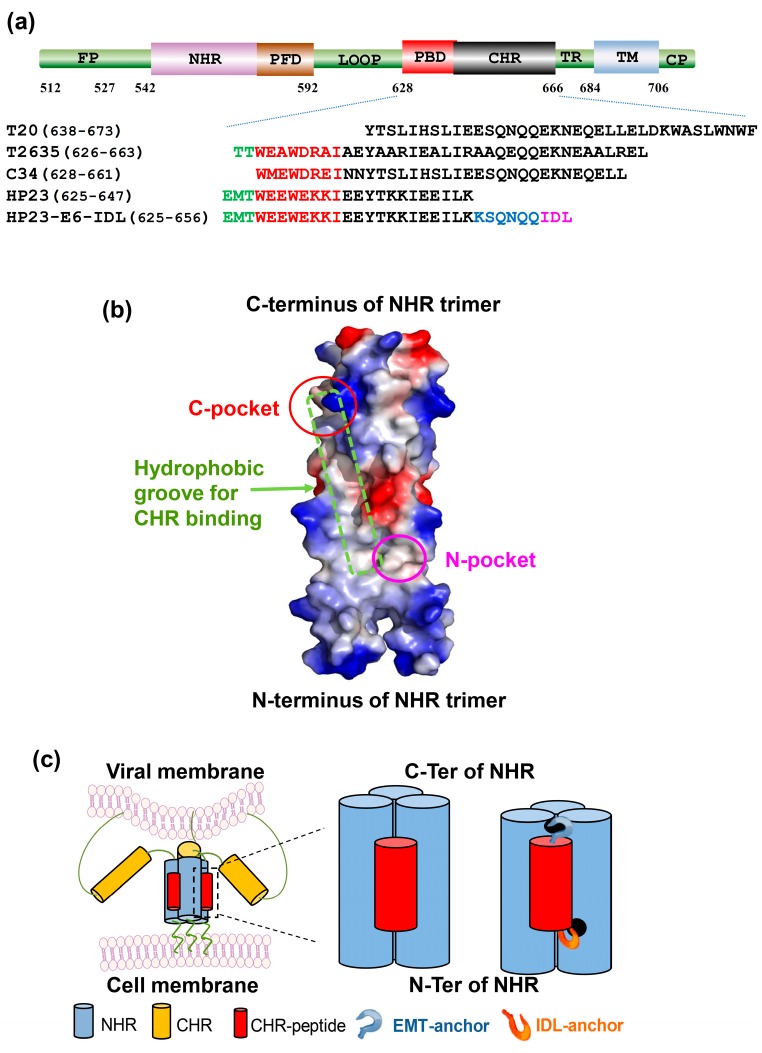
Design strategy for HP23-E6-IDL. (**a**) Domain distribution of HIV-1 gp41 and sequences of peptides. CP, cytoplasm region; TM, transmembrane region; TR, tryptophan-rich region; CHR, C-terminal heptad repeat; PBD, pocket-binding domain; PFD, pocket-forming domain; NHR, N-terminal heptad repeat; FP, fusion peptide region; (**b**) Crystal structure of gp41 NHR trimer (PDB 2X7R) is shown as an electrostatic surface. Red and pink circles indicate the NHR C-terminal deep hydrophobic pocket and N-terminal shallow pocket, respectively. Green box indicates the hydrophobic groove for CHR binding; (**c**) Schematic diagram showing the design concept of HP23-E6-IDL. HP23-E6-IDL with both EMT-anchor and IDL-anchor is expected to bind to the NHR more stably and block the formation of a homologous 6-helix bundle, thereby inhibiting viral infection with increased potency and genetic barrier to resistance.

HP23, the shortest CHR peptide (23 aa), has relatively potent anti-HIV-1 activity, mainly because its N-terminal portion contains the EMT-anchor structure and pocket-binding sequence, which can strongly bind to the deep hydrophobic pocket (C-pocket) in the C-terminal groove of the NHR-trimer (Figure 1b,c). However, HIV-1 with E49K mutation in the gp41 NHR became resistant to HP23 [24]. In our previous study, we identified a shallow pocket (N-pocket) in the N-terminal region of the NHR domain (Figure 1b) and found that addition of an IDL-anchor to the C-terminus of a CHR-peptide could significantly improve the peptide’s anti-HIV-1 potency and resistance profile (Figure 1c) [27]. Therefore, we proposed that the addition of an IDL-anchor to the C-terminus of HP23 could result in more stable binding of this peptide with the groove on the NHR-trimer since its N- and C-terminal anchors could bind to the C- and N-pockets on the NHR-trimer, respectively (Figure 1c). By analyzing the crystal structure of the NHR trimer core in the 6-HB, we noticed that the HP23 sequence must be extended using six amino acids at its C-terminus in order to allow the added IDL anchor to bind to the N-pocket. Therefore, we designed a new peptide, designated as HP23-E6-IDL, by extending by six amino acids and adding the IDL sequence (Figure 1a).

**Figure 2:**
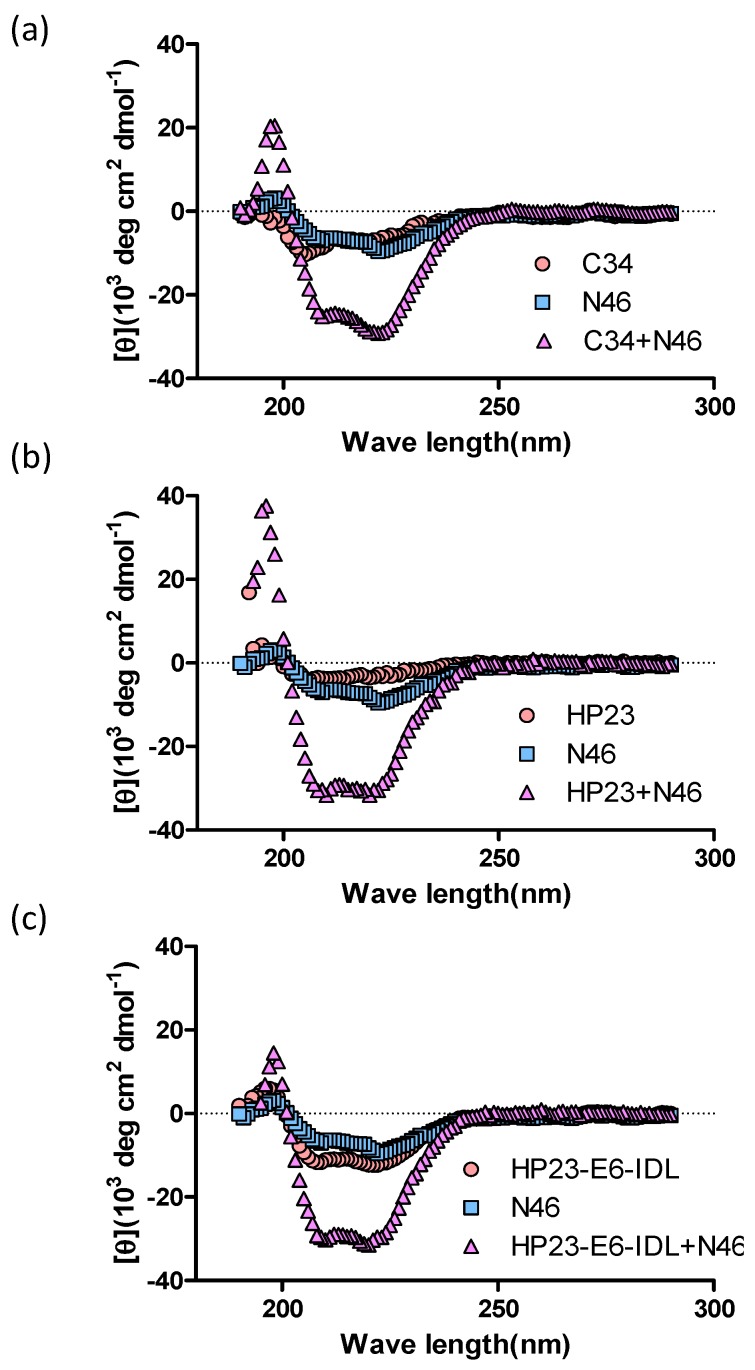
Secondary structures of CHR peptide-NHR peptide complexes. (**a**–**c**) Circular dichroism (CD) spectroscopy results for the complexes formed by N46 and C34, HP23, and HP23-E6-IDL are shown. The circular dichroism spectra of these complexes displayed typical double minima at 208 and 222 nm for the α-helical feature. C34 was used as a positive control.

We first analyzed the secondary structure of the complex formed by NHR peptide N46 (aa 536-581 of NHR) and HP23-E6-IDL (HP23 and C34 as controls) by circular dichroism (CD) spectroscopy. As shown in Figure 2, HP23-E6-IDL, like C34 and HP23, could interact with N46 to form an α-helical structure, suggesting that HP23-E6-IDL could form a stable 6-HB structure with NHR of the HIV-1 gp41.

**Figure 3:**
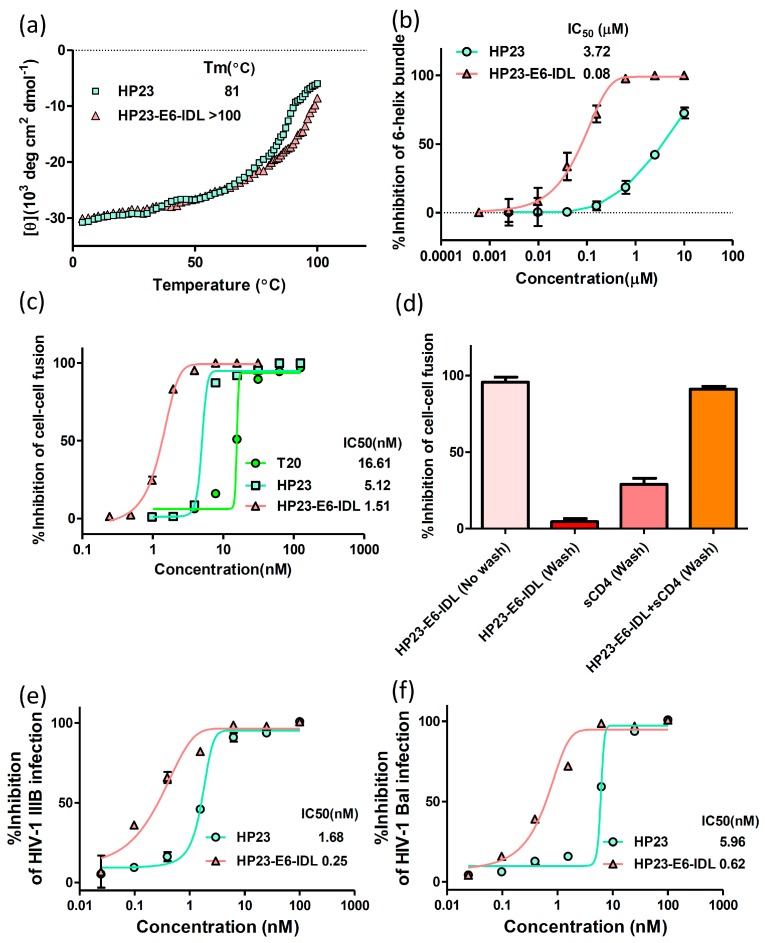
Inhibitory activity of HP23-E6-IDL. (**a**) Comparison of melting curves of the complexes formed by N46 and HP23 or HP23-E6-IDL. HP23-E6-IDL shows better thermostability; (**b**) Inhibitory activities of HP23 and HP23-E6-IDL against 6-HB formation between N46 and C34; (**c**) Inhibitory activities of peptides against cell–cell fusion between H9/HIV-1IIIB cells and MT-2 cells; (**d**) An HIV-1 Env-mediated cell–cell fusion washout assay was performed to determine the Env engagement of CD4-receptor in the HP23-E6-IDL-mediated inhibition of HIV-1 entry. Inhibition rates were as follows: HP23-E6-IDL alone without wash, 95.8%; HP23-E6-IDL alone with wash, 4.6%; sCD4 alone with wash, 28.9%; HP23-E6-IDL and sCD4 with wash, 91.1%; (**e**) Inhibitory activities of HP23 and HP23-E6-IDL against HIV-1 IIIB (X4 virus) infection; (**f**) Inhibitory activities of HP23 and HP23-E6-IDL against HIV-1 Bal (R5 virus) infection. Error bars in this figure show standard deviations.

Next, we performed a thermal denaturation experiment to compare the thermostability (*T*_m_ value) of 6-HBs formed between N46 and HP23-E6-IDL or HP23. As shown in Figure 3a, the *T*_m_ value of 6-HB formed between N46 and HP23 is 81 °C, while that formed by N46 and HP23-E6-IDL is beyond 100 °C. These results indicate that the binding affinity between N46 and HP23-E6-IDL is much higher than that between N46 and HP23.

We then compared the inhibitory activity of HP23 and HP23-E6-IDL on 6-HB formation by N46 and C34 by using the NC-1 monoclonal antibody which specifically binds to the 6-HB formed by N36 or N46 and C34 in ELISAs. As shown in Figure 3b, HP23-E6-IDL exhibited about 47-fold stronger inhibition compared to HP23 on 6-HB formation, suggesting that HP23-E6-IDL has greater affinity for N46 compared to HP23.

### 2.2. HP23-E6-IDL Inhibited HIV-1 Env-Mediated Cell–Cell Fusion

Having proved that HP23-E6-IDL has high affinity towards NHR, we then tested its inhibitory activity on HIV-1 Env-mediated cell–cell fusion. In this test, MT-2 cells that express the receptor CD4 and the co-receptor CXCR4 were used as target cells while H9/HIV-1_IIIB_ cells that stably express HIV-1 Env protein were used as the effector cells. H9/HIV-1IIIB cells fused with MT-2 cells in the absence of fusion inhibitors. However, as shown in Figure 3c, HP23-E6-IDL blocked this fusion process in a dose-dependent manner. The IC_50_ of HP23-E6-IDL was 1.51 nM, which is 3.4- and 11-fold more potent than HP23 and T20, respectively.

We also performed HIV-1 Env-mediated cell–cell fusion washout assays to determine whether peptide-mediated inhibition depends on Env engagement of the CD4 receptor (Figure 3d). HP23-E6-IDL could inhibit HIV-1-mediated membrane fusion when it was incubated with H9/HIV-1IIIB cells before the addition of MT-2 cells. When the mixture of HP23-E6-IDL and H9/HIV-1IIIB cells was washed before the addition of MT-2 cells, HP23-E6-IDL ceased its inhibitory activity against membrane fusion. However, when sCD4 was added to the mixture of HP23-E6-IDL and H9/HIV-1IIIB cells before washing, the fusion between H9/HIV-1IIIB cells and MT-2 cells was blocked. These results suggest that HP23-E6-IDL can only bind to the NHR domain of gp41 and inhibit HIV-1 entry if gp120 is occupied by CD4. That means that only when gp41 is in the prehairpin conformation induced by gp120 after its binding to CD4, HP23-E6-IDL can bind to the NHR domain of gp41 and block the viral gp41 6-HB formation, thus resulting in inhibition of HIV-1 fusion with and entry into the target cell.

### 2.3. HP23-E6-IDL Exhibited Potent Antiviral Activity Against a Broad Spectrum of HIV-1 Strains, Including Those Resistant to T20, T2635, and HP23

We further compared the inhibitory activities of HP23 and HP23-E6-IDL on HIV-1 laboratory-adapted strains. As expected, compared to HP23, HP23-E6-IDL exhibited more potent antiviral activity against the HIV-1 strains tested (Figure 3e,f). The IC_50_ values of HP23-E6-IDL against HIV-1 X4 (IIIB) and R5 (Bal) strains are 0.25 and 0.62 nM, respectively, representing about 6.7- and 9.6-fold more potency than HP23.

We then tested the antiviral ability of HP23-E6-IDL against a panel of HIV-1 clinical isolates. As shown in Table 1, HP23-E6-IDL had potent inhibitory activity against all the clinical isolates with different subtypes tested, and its IC_50_s ranged from 0.1 to 1.6 nM, values that are, on average, about 6.1-fold more potent than HP23.

Most importantly, HP23-E6-IDL could inhibit infection by all resistant mutants tested with IC_50_s ranging from 0.3 to 5.2 nM. For HIV-1 T2635- (Table 2), T20- and HP23-resistant strains (Table 3), HP23-E6-IDL was about 5.6-, 5.4- and >12-fold more potent than HP23, respectively. These results suggest that HP23-E6-IDL can bind more tightly to the NHR domain of HIV-1 gp41 than HP23, making it a good candidate for further development as a new HIV-1 drug.

## 3. Discussion

HIV-1 mortality and incidence have been dramatically reduced because of the global scale-up of antiretroviral therapy (ART). However, the continuous emergence of drug-resistant HIV-1 mutants calls for the development of new anti-HIV drugs with improved drug-resistant profiles or higher genetic barrier to resistance. Here, we designed a new conjugated peptide, which consists of HP23, E6-linker, and IDL-anchor. HP23-E6-IDL can bind to the NHR domain of gp41 when gp41 is in the prehairpin conformation induced by gp120 binding to CD4, thus inhibiting the formation of 6-HB and blocking HIV-1 entry into the target cell. HP23-E6-IDL exhibited, on average, about 6.7-fold more potency than HP23 against all 36 HIV-1 strains tested, including all mutants with resistance to T20, T2635, and/or HP23.

In previous studies of T2653-resistant HIV-1 variants, it was surprising to find that only a few mutations occurred in the assumed binding sites of T2635 in the NHR domain [23], while so many more mutations could be found in the regions beyond the binding sites of NHR. For example, substitution N126K in the gp41 CHR domain has been reported to cause resistance to several HIV-1 drugs [15,28,29,30]. This mutation may result in accelerated 6-HB formation [12], thus shortening the window of opportunity for CHR peptides to bind the viral gp41 NHR domain. Mutations at position 126 in gp41 are associated with increased thermal stability and free energy of 6-HB [12]. Here, we found that HP23-E6-IDL could still inhibit HIV-1 variants with N126K, Q79E/N126K, K90E/N126K, E49K/N126K, and D36G/E49K/N126K mutations, proving its high affinity to the prehairpin intermediate of gp41.

In addition, mutations that weaken gp120-gp41 interactions may also accelerate 6-HB formation and cause resistance to CHR peptides. For example, Q79E and K90E mutations are located in the gp41 loop domain known to interact with gp120 [31,32]. Viruses with these mutations are resistant to T2635. However, HP23-E6-IDL is still effective against these HIV-1 mutants. Mutations in the FP domain of gp41 may also affect the speed and efficiency of FP insertion into the target membrane, which occurs prior to 6-HB formation [33]. Mutations at positions 3 and 6 in the FP domain were shown to confer resistance to a number of CHR peptides. However, once again, HP23-E6-IDL maintained its potent inhibitory activity against these variants.

Among all the resistant mutations in the gp41 of the 19 HIV-1-resistant strains we tested, only L34S and D36G mutations are located in the target pocket of the IDL-anchor. Interestingly, however, HP23-E6-IDL can still potently inhibit infection by strains with these mutations. It was reported that resistance of the CHR peptide-based HIV-1 fusion inhibitors may result from the shortened window period of opportunity provided by the prehairpin intermediate of gp41, rather than the reduced binding affinity to the mutant sites [12]. However, even though the affinity of HP23-E6-IDL to the mutated NHR is relatively low compared to the wild-type NHR sequence, we speculate that it is still high enough to bind to the NHR trimer with the shortened window period of gp41’s prehairpin intermediate, thus enabling inhibition of HIV-1 fusion with and entry into the target cell.

Our previous study has shown that addition of an MT anchor and an IDL anchor to the N- and C-termini of the peptide WQ, respectively, results in generation of a new peptide, designated MT-WQ-IDL, with significantly improved inhibitory activity against HIV-1 Bal infection (IC_50_ = 0.6 nM) [27]. It was reported that addition of a glutamic (E) residue to the N-terminus of a CHR-peptide with MT-hook could stabilize the MT hook structure [21]. We thus expected that HP23-E6-IDL, which contains the EMT-anchor, might have more potent anti-HIV-1 Bal infection than MT-WQ-IDL. Interestingly, we found that although HP23-E6-IDL had equal potency against HIV-1 Bal infection (IC_50_ = 0.6 nM) as that of MT-WQ-IDL, HP23-E6-IDL was generally more effective than MT-WQ-IDL against T20- and T2635-resistant strains [27]. These results suggest that addition of the EMT-anchor to a CHR-peptide is more favorable than addition of MT-anchor for designing CHR-peptide with improved antiviral activity against HIV-1 mutants resistant to CHR-peptides. Besides the low genetic barrier to resistance, the other advantages of T20 for clinical use are its short half-life and lack of oral bioavailability. Therefore, it is essential to further modify HP23-E6-IDL via different approaches, such as D-amino acid replacement [34], hydrocarbon stapling [35], glycosylation [36], PEGylation [37], and lipidation (e.g., addition of cholesterol or sphinganine to the peptide) [38]. By applying these modifications, the modified HP23-E6-IDL peptide is expected to have improved oral bioavailability and extended in vivo half-life.

Recently, broad neutralizing antibodies (bNAbs) have been recognized as potential anti-HIV therapeutics for treatment and prevention of HIV-1 infection [39]. However, bNAbs can also induce drug resistance [40,41,42,43]. Furthermore, bNAbs are much less effective in blocking HIV-1 cell–cell transmission than inhibiting replication of free virions [44]. Meanwhile, we have reported that combinations of CHR peptides with bNAbs exhibit synergistic activity against both drug-sensitive and -resistant HIV-1 strains [26,45]. Therefore, we expect that the combination of new peptide HP23-E6-IDL, with higher genetic barrier to resistance, and bNAbs may also have synergistic activity against HIV-1 mutants that are resistant to HIV entry inhibitors and bNAbs, thus reducing the dosage of anti-HIV drug used and the attendant cost.

## 4. Materials and Methods

### 4.1. Peptides

Peptides (Figure 1a) were synthesized by Synpeptide Co., Ltd. (Shanghai, China) with purity >95% as described previously [46]. Concentrations of the peptides in PBS were measured by NanodropTM 2000 Spectrophotometers (Thermo Fisher Scientific Inc., Waltham, MA, USA) as described previously [45] and calculated based on a theoretical molar-extinction coefficient according to the peptide sequences.

### 4.2. Circular Dichroism (CD) Spectroscopy

The secondary structure of the complexes of the NHR-peptide, N46, and a CHR-peptide was assessed by CD spectroscopy as described previously [47]. Briefly, N46 and CHR-peptide were solubilized together in ddH_2_O with final concentration as 10 μM. The mixture was incubated at 37 °C for 30 min and then tested on a Jasco spectropolarimeter (Model J-815; Jasco, Inc., Easton, MD, USA), using a 1-nm bandwidth with a 1-nm step resolution from 195 to 260 nm at room temperature. The baseline curve was determined on ddH_2_O alone. The helical content was the ratio of (the mean residue ellipticity at 222 nm)/(the value expected for 100% helix formation) (−3.3 × 10^−4^ deg cm^2^ dmol^−1^).

The thermal denaturation experiment was performed by monitoring the ellipticity change at 222 nm with increasing temperature (4–98 °C). The temperature was increased at a rate of 1.2 °C/min; data were acquired at 222 nm at a frequency of 0.25 Hz at a 1-nm bandwidth. The melting temperature (*T*_m_) was calculated by the Denatured Protein Analysis program (Jasco, Inc., Easton, MD, USA).

### 4.3. Inhibition of 6-HB Formation by Peptides In Vitro

The inhibitory activity of peptides on 6-HB formation was measured by a modified ELISA as previously described [48]. Briefly, a 96-well polystyrene plate (Costar, Corning Inc., Corning, NY, USA) was coated with 50 μL 2 μg/mL NY364 (a polyclonal antibody for HIV-1 gp41 subunit) in 0.1 M Tris buffer (pH 8.8). A mixture of 1 μM N46 and C-peptide with graded concentrations was added to the coated plate after incubation at 37 °C for 30 min. After another 1 hour incubation at 37 °C, the plate was washed with washing buffer (PBS containing 0.1% Tween 20) three times and refilled by 50 μL 1 μg/mL NC-1 (a monoclonal antibody specific for HIV-1 gp41 6-HB formed by NHR and CHR) [49]. Afterwards, 50 μL of horseradish peroxidase (HRP)-labeled rabbit anti-mouse antibody (Sigma, St. Louis, MO, USA) (1:4000 diluted) were added to the wells of plate, followed by incubation for 1 h and washing. Finally, the substrate 3,3,5,5-tetramethylbenzidine (TMB, Sigma, St. Louis, MO, USA) was added. Absorbance at 450 nm (A450) was tested by an ELISA reader (Ultra 384, Tecan, NC, USA).

### 4.4. HIV-1-Mediated Cell–Cell Fusion Assay

A dye transfer assay was used to detect HIV-1 Env-mediated cell-cell fusion as described previously [50]. One mL of 2 × 10^4^/mL H9/HIV-1IIIB cells labeled with 2.5 μL of 1 nM fluorescent reagent, Calcein AM (Molecular Probes, Inc., Eugene, Oregon), was incubated at 37 °C for 30 min. For each well of the 96-well plate, 50 μL 2 × 10^4^/mL labeled H9/HIV-1IIIB cells were then incubated with 100 μL 1 × 10^5^/mL MT-2 cells at 37 °C for 2 h in the presence or absence of the tested peptide at graded concentrations. The fused and unfused Calcein-labeled HIV-1 IIIB cells were counted under an inverted fluorescence microscope (Zeiss, Oberkochen, Germany). The IC_50_ values were calculated by using the Calcusyn computer program (Biosoft, Ferguson, MO, USA).

An HIV-1 Env-mediated cell-cell fusion washout assay was conducted as previously described [51]. Briefly, HP23-E6-IDL (5 nM), sCD4 (100 nM), and HP23-E6-IDL/sCD4 mixture, respectively, were pre-incubated with H9/HIV-1IIIB cells labeled with Calcein AM at 37 °C for 0.5 h. The treated H9/HIV-1IIIB cells were washed with PBS (unwashed H9/HIV-1IIIB cells with HP23-E6-IDL were taken as control) before addition of MT-2 cells. After incubation at 37 °C for 2 h, the fused and unfused Calcein-labeled HIV-1 IIIB cells were counted as described above.

### 4.5. Inhibition of HIV-1 Infection by Peptides

Inhibitory activities of peptides on infection by laboratory-adapted HIV-1 X4 strain IIIB and HIV-1 R5 strain Bal, and HIV-1 clinical strains, as well as T20-, T2635- and HP23-resistant strains, were determined as described previously [52]. For each well of the 96-well plate, 104 MT-2 cells or CEMx174 5.25 M7 cells with graded concentrations of peptide were infected by 100 μL TCID50 of the HIV-1 virus X4 or R5 strains, respectively. After overnight culture, the medium was replaced with fresh RPMI 1640 medium containing 10% FBS. Fifty microliters of culture supernatant were collected from each well on the fourth day for MT-2 cells and the seventh day for CEMx174 5.25 M7 cells. The supernatant was mixed with equal volumes of 5% Triton X-100. The p24 antigen was detected by ELISA as previously described [53]. IC_50_ values were calculated using the Calcusyn software program (Biosoft, Ferguson, MO, USA).

## Figures and Tables

**Table 1:** Inhibitory activities of HP23 and HP23-E6-IDL against HIV-1 clinical isolates (subtype, tropism).

Viruses	IC_50_ (nM)	
	HP23	HP23-E6-IDL
92UG029 (A, X4)	1.6 ± 0.3	0.8 ± 0.4
KER2018 (A, R5)	1.9 ± 0.2	0.8 ± 0.1
KNH1135 (A, R5)	2.2 ± 0.3	0.5 ± 0.2
US4GS007 (B, R5)	3.9 ± 0.6	0.8 ± 0.1
BK132/GS009 (B, X4)	7.2 ± 0.5	0.7 ± 0.2
90US_873 (B, R5)	3.4 ± 0.6	1.0 ± 0.1
93IN101 (C, R5)	3.7 ± 0.3	0.6 ± 0.1
92UG024 (D, X4)	3.5 ± 0.2	0.6 ± 0.1
93BR020 (F, X4/R5)	4.5 ± 0.2	1.0 ± 0.2
BCF02 (O, R5)	9.4 ± 0.8	1.6 ± 0.2
92TH009 (A/E, R5)	0.8 ± 0.2	0.1 ± 0.1
NP1525 (A/E, X4/R5)	9.5 ± 0.6	0.6 ± 0.2

**Table 2:** Inhibitory activities of HP23 and HP23-E6-IDL against HIV-1 T2635-resistant strains.

Viruses	IC_50_ (nM)	
	HP23	HP23-E6-IDL
wild-type	2.8 ± 0.2	1.5 ± 0.2
H3C	5.4 ± 0.4	0.7 ± 0.2
A6V	1.6 ± 0.5	0.3 ± 0.1
Q66R	2.8 ± 0.1	0.4 ± 0.2
Q79E	4.2 ± 0.7	1.3 ± 0.2
K90E	9.4 ± 0.3	2.2 ± 0.2
N113E	2.3 ± 0.5	0.7 ± 0.1
N126K	8.6 ± 0.4	1.6 ± 0.2
K154Q	7.4 ± 0.4	1.5 ± 0.2
Q79E/N126K	5.2 ± 0.4	0.7 ± 0.1
K90E/N126K	4.2 ± 0.3	0.3 ± 0.1
Q66R/N113E	3.6 ± 0.3	1.1 ± 0.1

**Table 3:** Inhibitory activities of HP23 and HP23-E6-IDL against HIV-1 T20- and HP23-resistant strains.

Viruses	IC_50_ (nM)	
	HP23	HP23-E6-IDL
T20-resistant strains		
WT	3.1 ± 0.1	1.2 ± 0.2
V38A	2.1 ± 0.7	0.5 ± 0.2
V38A, N42D	2.9 ± 0.3	1.5 ± 0.2
V38E, N42S	6.2 ± 0.3	0.6 ± 0.1
V38A, N42T	1.5 ± 0.3	0.3 ± 0.1
HP23-resistant strains		
wild-type	3.3 ± 0.3	0.6 ± 0.1
E49K	13.4 ± 0.7	1.9 ± 0.2
E49K/N126K	53.8 ± 1.2	2.6 ± 0.3
D36G/E49K/N126K	>60	4.0 ± 0.4
L34S/D36G/E49K/E136G	>60	5.2 ± 1.1
